# Analysis of the Dynamic Relationship between Green Economy Efficiency and Urban Land Development Intensity in China

**DOI:** 10.3390/ijerph19137960

**Published:** 2022-06-29

**Authors:** Jiao Hou, Xinhai Lu, Shiman Wu, Shangan Ke, Jia Li

**Affiliations:** 1 College of Public Administration, Central China Normal University, Wuhan 430079, China; houjiao@mail.ccnu.edu.cn (J.H.); keshangan@126.com (S.K.); lijia-wh@mails.ccnu.edu.cn (J.L.); 2 School of Management, Wuhan Institute of Technology, Wuhan 430205, China; wushiman314@foxmail.com

**Keywords:** green economic efficiency, urban land development intensity, interactive response

## Abstract

The improvement of green economic efficiency (GEE) should be realized under reasonable urban land development intensity (ULDI). Improving GEE can also help alleviate the negative externalities of excessive or unreasonable ULDI. Clarifying the interactive response mechanism between GEE and ULDI is a key link in regional sustainable development. Therefore, this paper uses the super-efficiency slack-based model (SBM) method, panel entropy method, and panel vector auto regression model to comprehensively analyze the interactive response relationship between GEE and ULDI in 283 prefecture-level cities in China from 2003 to 2019. This paper finds that: (1) during the research period, both the GEE and ULDI showed a relatively obvious upward trend, which is manifested in the fact that ULDI increased year by year while GEE overall increased in volatility. The growth and evolution trend of ULDI and GEE has the characteristics of interaction and coordination; (2) there is a two-way interactive Granger causality between ULDI and GEE, showing a positive interactive response effect; and (3) both ULDI and GEE have positive inertial growth and self-enhancement mechanisms. In the long run, GEE has a greater impact on the change of ULDI.

## 1. Introduction

The complex interaction between economic development and urban land use has always been the focus of global sustainable development. Although there is a big gap between developed and developing countries [1], they are both faced with the problem that economic growth is not sustainable because of the rigid restriction of the total amount of urban land resources [2]. The whole world chooses to implement green economic development and higher intensity urban land use to solve the unsustainable problem of the traditional economic development model. Since the 20th century, China’s economic development achievements have attracted worldwide attention. However, the reality is that China’s traditional economic development model of high pollution and extensive utilization still exists [3,4]. Economic development is strongly dependent on the development and utilization of land resources [5], and the problems of high-intensity or unreasonable urban land use, such as the disorderly expansion of urban construction land, structural imbalance, and overall low level of resource allocation efficiency, are becoming increasingly prominent [6,7]. Realizing the benign interaction between urban land development and green economic development is a strategic task and an important path for China, which is in the transition stage to green and low-carbon development, both at present and for a long time in the future. At the same time, studying the interaction between green economic development and urban land use intensity, and summarizing China’s experience in the above aspects, can provide path reference and experience for other developing countries to achieve sustainable development.

Green economic efficiency (GEE) reflects the economic efficiency of a country or region after comprehensive consideration of resource depletion and environmental impact, and is directly related to the green economy development in the future [8]. In the research on the measurement of GEE, existing studies incorporated the cost of environmental pollution control into the production function as an undesired output, and used the Solow residual value method to measure green economy productivity to judge whether a country or region could achieve green economic development [9,10,11]. With the continuous deepening of the index system and method innovation research on GEE, scholars have selected indicators based on the traditional indicator system of labor, capital, resource input, economic expected output, and environmental pollution discharge as undesired output [12], adding consideration for the input and output of factors such as technology and smog [13]. The parametric method and the non-parametric method are two methods to measure green economy efficiency. Among them, the parameter method is used to measure the GEE by setting the specific form of the production function, and the more commonly used method is the stochastic frontier analysis method (SFA) [14]. Nonparametric methods mainly refer to data envelopment analysis (DEA) and its derived models [15,16]. The DEA model can measure the GEE under multiple input factors and multiple input–output conditions, but it can easily lead to biased results because it cannot consider the influence of input–output slack variables. In recent years, scholars have mostly used the slack-based model (SBM) based on slack variables proposed by Tone (2001) [17] and the super-efficient SBM model that can make up for the SBM model to measure the efficiency value of multiple decision-making units with a unit of 1 [18].

Urban land development intensity (ULDI) is an index to comprehensively evaluate the status of urban land development and utilization, including the comprehensive characterization of the urban land development scale, urban land development benefits, and urban land development structure [19,20,21,22]. In the above definition, the actual performance of urban land development is the expansion of the scale of construction land and the reduction in cultivated land, forest land, water areas, etc. [23,24]. In this process, the same urban land development scale produces different functions and benefits because of different urban land-use structures [25]. Scholars in various countries have reached a general consensus that the ULDI is an important part of the urban space management and control system, and its scientific measurement and evaluation analysis is an important path to optimize the urban land development pattern [19,26]. Under the background of this theoretical research, Chinese scholars have achieved quite rich results in the concept and connotation measurement of ULDI. There are usually two research approaches. The first is to use a single indicator, such as the proportion of regional construction land area to regional land area [27], building density [28], compactness [29], and plot ratio [30,31] to measure ULDI. In recent years, scholars mainly directly use the proportion of the construction land area in the urban area to the total land area in the urban area to measure the urban land development intensity [32]. The second is to construct an index system from multiple levels for comprehensive evaluation according to the characteristics of urban land development. For example, Wang et al. [33] selected indicators from six aspects including construction land development intensity, population density, economy, ecological environment, infrastructure, and public service facility intensity to comprehensively evaluate the ULDI of typical Chinese cities. Liu Yanjun et al. [34] constructed a theoretical analysis framework for ULDI including the level and extent of construction land use, population, and socio-economic bearing intensity in urban areas, and selected corresponding indicators from three aspects of quantity, structure, and benefit to measure ULDI in northeast China. Kong Xuesong et al. [35] selected indicators from three aspects of urban land development density, development benefit, and development degree to measure and evaluate the ULDI of county-level units in Jiangsu Province.

Existing studies have explored the interaction between ULDI and GEE. Some scholars regard construction land scale and land-use structure changes as the dominant characteristics of urban land development [36,37] and focus on discussing the economic, social, and ecological benefits brought about by construction land scale changes and different urban land-use structures [38,39]. Changes in the scale of urban construction land can have a positive impact on the quality of the economy, society, and ecological environment in the development of green economy, but the rapid expansion of urban construction land has adversely affected the urban ecological environment and the lives of urban residents, thereby reducing the GEE [40]. At the same time, the changes in social structure, economic structure, and ecological structure caused by green development economy will ultimately be reflected in the urban land-use structure and its changes [41,42]. Some scholars have also studied the impact of green economic development on the ULDI. Factor agglomeration and efficient allocation are typical features of GEE improvement [43]. The agglomeration economy, technological innovation and investment expansion caused by the agglomeration of factors and the efficient allocation of resources significantly affect the ULDI. Scholars generally agree that industrial agglomeration, industrial structure upgrading, and technological innovation are the direct reasons that affect the optimization of urban land development [44,45,46]. However, with the continuous agglomeration of factors, there may be a “crowding effect”, resulting in problems such as the intensification of the contradiction between man and land and the blind expansion of construction land [47]. Scholars are also concerned about environmental regulation as an effective means for green economic development to influence urban land development [48]. Environmental regulation plays a certain role in alleviating excessive or unreasonable urban land development through structural effects, innovation effects, spillover effects, etc., and improves the efficiency of resource allocation, thereby affecting the intensity of urban land development [49,50].

There is an interaction and mutual influence between ULDI and GEE. The optimization of urban land development can promote green economy development, and economic transformation and green development can also control the total scale of urban land development and optimize the pattern of urban land development. However, the existing research mainly studies the one-way effect of GEE on ULDI or ULDI on GEE. These studies have not given the dynamic relationship between GEE and ULDI, and the research on the interaction and response mechanism between GEE and ULDI is still insufficient. Therefore, the contribution of this paper is to construct the evaluation index system of GEE and ULDI, respectively, and expand the depth and breadth of existing research on the relationship between GEE and ULDI. This paper will take 283 prefecture-level and above cities in China as the research objects and select the sample data from 2003 to 2019. First, the paper uses the super-efficiency SBM model and the panel entropy method to measure GEE and ULDI, respectively, reveal their evolutionary characteristics, and preliminarily determine the relationship between the two. Second, the paper uses the panel vector autoregression (PVAR) model to explore the dynamic interaction mechanism between the two. Finally, policies and suggestions are put forward to better realize the good interaction and sustainable development between ULDI and GEE.

## 2. Methods and Data

### 2.1. GEE Evaluation Model

#### 2.1.1. The Super-Efficiency SBM Model

We adopt the super-efficiency SBM model based on undesired output to measure GEE. Charnes and Cooper (1990) [51] first proposed the data envelopment analysis (DEA), and then in 2001, Tone [17] proposed a non-radial slacks-based measure (SBM) model based on the traditional DEA model. In 2002, Tone proposed a super-efficiency SBM model based on the non-radial SBM model with modified slack variables [52]. The super-efficiency SBM model can not only consider slack variables and avoid the bias caused by the selection of radial and angular selections, but also further rank effective research units with an efficiency value greater than or equal to 1. The super-efficiency SBM model that incorporates undesired outputs is widely used by scholars to measure efficiency. It is of great significance especially in the study of ecological efficiency in economic development and economic development efficiency under the constraints of resources and environment [53]. The calculation model is:

(1) $$\min\rho_{SE}=\frac{\frac{1}{m}\sum_{i=1}^{m}\left(\bar{x}/x_{ik}\right)}{\frac{1}{r_{1}+r_{2}}\left(\sum_{j=1}^{r_{1}}\bar{y^{d}}/y_{jk}^{d}+\sum_{q=1}^{r_{2}}\bar{y^{u}}/y_{qk}^{u}\right)}$$

(2) $$\left\{ \begin{array}{l} \bar{x}\geq \sum_{j=1,\neq k}^{n}x_{ij}\lambda_{j};\bar{y^{d}}\leq \sum_{j=1,\neq k}^{n}y_{sj}^{d}\lambda_{j};\\ \bar{y^{d}}\geq \sum_{j=1,\neq k}^{n}y_{qj}^{d}\lambda_{j};\bar{x}\geq x_{k};\\ \bar{y^{d}}\leq y_{k}^{d};\bar{y^{u}}\geq y_{k}^{u};\\ \lambda_{j}\geq 0,i=1,2,\ldots ,m;j=1,2,\ldots ,n;\\ s=1,2,\ldots ,r_{1};q=1,2,\ldots ,r_{2}; \end{array}\right.$$

In the formula, assume there are n decision making units (DMUs). Each DMU has m inputs, $r_{1}$ expected outputs, and $r_{2}$ undesirable outputs. x is the elements in the input matrix. $y^{d}$ is the elements in the desired output matrix. $y^{u}$ is the elements in the undesired output matrix. ρ is the GEE value obtained from the measure.

#### 2.1.2. Selection of Indicators

Based on the existing research results, the GEE measurement index system is constructed from three aspects: input, expected output, and undesired output (Table 1).

The input factors in GEE include non-resource input factors and resource input factors. Non-resource input factors mainly consider labor, capital, and technology input. We select the number of employees and capital stock as the corresponding index to measure labor input and capital input. Green technology innovation is an effective way for China’s economic transformation to green development to achieve sustainable development goals [54]. In existing research, R&D funding is an index to measure technology input in economic efficiency [55], but not all R&D funding goes to green innovation. Patent applications can reflect the progress of technological innovation [56], among which the green patent number can be used to evaluate the field of green technological innovation [57,58]. Therefore, this paper chooses the number of green patents as an indicator to measure technology input in GEE. The resource input element is mainly represented by the indicator of electricity consumption in the whole society.

The expected output is expressed by the indicator of GDP. The undesired output is generally represented by the comprehensive index of industrial pollution and, taking into account the current “dual carbon” goal vision, carbon emissions are also included in the undesired output.

### 2.2. ULDI Evaluation Model

#### 2.2.1. Panel Entropy Method

We adopt the panel entropy method to measure ULDI. The entropy method determines the indicator weight according to the size of the information provided by the indicator observations. Entropy is derived from the physical concept of thermodynamics and was introduced to information theory by Shannon in 1948. In information theory, entropy is used to measure uncertainty. The smaller the amount of information, the greater the uncertainty, and the greater the entropy [59]. Based on this characteristic, the entropy value can be calculated to determine the degree of dispersion of an index. The greater the degree of dispersion of the index, the greater the impact on the comprehensive evaluation of the comprehensive index, and the greater the weight given to the index. The entropy value method can determine the index weight according to the degree of variation of the index value, which can avoid the lack of objectivity that is due to the subjective judgment in the subjective weighting method, and can also avoid the lack of information in the principal component analysis method. The entropy value method applied in practice is the most extensive. Moreover, the traditional entropy method to determine the weight can only deal with cross-sectional data, which makes it difficult to compare between different years. In this paper, the time variable is introduced into the improved panel entropy method to assign the index weight. The calculation model is:

The first step is to select indicators. There are m city t years n indicators, then $x_{ijk}$ represents the value of the k-th index in the j-th year of the i-th city. In this paper, $x_{ijk}$ is the index selected to judge the ULDI.

The second step is to standardize the indicators. Due to the differences in dimensions and units of different indicators, the extreme value method is selected to standardize the indicators. After the positive and negative indicators are determined, normalization is performed:

(3) $$x{’}_{ijk}=\frac{x_{ijk}-x_{\min k}}{x_{\max k}-x_{\min k}}$$

where $x_{\min k}$ represents the minimum values of the k-th index in the j-th year of the i-th city. $x_{\max k}$ represents the maximum values of the k-th index in the j-th year of the i-th city. The $x{’}_{ijk}$ obtained after the normalization of $x_{ijk}$ represents the relative size in m cities and t years, and the value is between 0 and 1.

The third step is to determine the indicator weight:

(4) $$y_{ijk}=x{’}_{ijk}/\sum_{i}\sum_{j}x{’}_{ijk}$$

The fourth step is to calculate the entropy value of the k-th indicator:

(5) $$e_{k}=-\frac{1}{\theta }\sum_{i}\sum_{j}y_{ijk}\ln(y_{ijk})$$

where the constant θ>0, and θ is only related to the number of samples m⋅t. We generally make θ=ln(m⋅t), then $0\leq e_{k}\leq 1$, ln is the natural logarithm.

The fifth step is to calculate the information utility value of the k-th indicator:

(6) $$g_{k}=1-e_{k}$$

The sixth step is to calculate the weight of the information utility value of the k-th indicator:

(7) $$w_{k}=(1-e_{k})/\sum_{k}(1-e_{k})$$

The seventh step is to calculate the comprehensive score of each city’s ULDI:

(8) $$H_{ij}=\sum_{k}w_{k}x{’}_{ijk}$$

#### 2.2.2. Selection of Indicators

Urban land development intensity is a comprehensive characterization of urban land development scale, urban land development benefit, and urban land development structure (Table 2).

The total scale of urban construction land is the main content of the scale of urban land development, which is represented by the ratio of the area of construction land in the region to the total land area of the region.

The purpose of urban land development is to produce economic, social, and ecological benefits through the development and utilization of land resources to meet the needs of human production and life. In terms of economic benefits, this paper selects the industrial non-agricultural rate and GDP per land to characterize the economic benefits of urban land development. The social benefits are reflected in the support of people’s income, settlement, and public services. Three indicators, namely per capita disposable income, per capita residential land area, and per capita road area, are selected. In terms of ecological benefits, regional green space is an important component of ecological space, which can provide ecological assistance for social and economic development by improving the ecological environment. This paper selects the green space per capita as an indicator.

In this paper, the information entropy index of construction land structure is selected as the index to judge the urban land development structure. The urban land development structure is reflected in various construction land types. The actual manifestation of urban land development is the expansion of the number and scale of construction land and the reduction in cultivated land, forest land, water areas, etc. With the changes in the degree of urban land development affected by human social and economic activities, the type of structure of construction land has been further significantly changed. The specific method is to first refer to the Standard for Classification of Urban Land and Planning and Construction Land (GB501372011) and determine that the construction land mainly includes eight types of land. Second, calculate the ratio of various types of construction land to the total area of regional construction land, expressed as $P_{1,2,\ldots ,8}$. Finally, according to the entropy formula $-\sum_{i=1}^{8}P_{i}\ln P_{i}$, the entropy value of the construction land structure information is calculated.

### 2.3. Panel VAR Model

We use the panel vector autoregression (PVAR) model to reveal the dynamic interaction mechanism between green economic efficiency and urban land development intensity. Based on the univariate autoregression (AR) model, Sims proposes a vector autoregression (VAR) model. The vector autoregression model is used for the prediction research of time series variables and the analysis of variables affected by random disturbances by realizing the regression analysis of the current variables on several lag variables of all variables. It is now widely used to analyze the dynamic correlation between multivariate time series variables. However, since the VAR model cannot handle long-term panel data, a PVAR model for panel data analysis was proposed. 

The previous studies on the interaction between GEE and ULDI above showed that the relationship between GEE and ULDI is complex. There is a mutual influence between GEE and ULDI, which means that endogenous causality may occur among GEE and ULDI. Therefore, this paper constructs a PVAR model to accurately identify the interaction and response mechanism between GEE and ULDI. The calculation model is:

(9) $$y_{it}=\alpha_{1}+\sum_{p=1}^{n}\beta_{p}y_{it-p}+\gamma x_{it}+\varepsilon_{it}$$

where $y_{it}$ is a multi-dimensional endogenous variable, which is GEE and ULDI; p is a lag period; $y_{it-p}$ is a lag period variable; $x_{it}$ is an exogenous variable; and $\varepsilon_{it}$ is the disturbance vector. The disturbance vector is only related to the current variable, independent of lag variables; α is the intercept term; and β and γ are the coefficient.

### 2.4. Research Samples and Data Sources

This paper takes prefecture-level cities in China as the research sample area. Considering the availability and completeness of data, the sample to be investigated in this paper is ultimately 283 prefecture-level and above cities in China. The study sample period is 2003–2019. Except for the green patent data from the CNRDS China Research Data Service Platform, other data related to index variables are all from the “China Urban Statistical Yearbook”, “China Urban Construction Statistical Yearbook”, statistical yearbooks over the years, and national economic and social development during the sample period. For very few cities, the missing data in some years were supplemented by interpolation.

## 3. Results

### 3.1. Evolutionary Characteristics of ULDI and GEE

**Figure 1:**
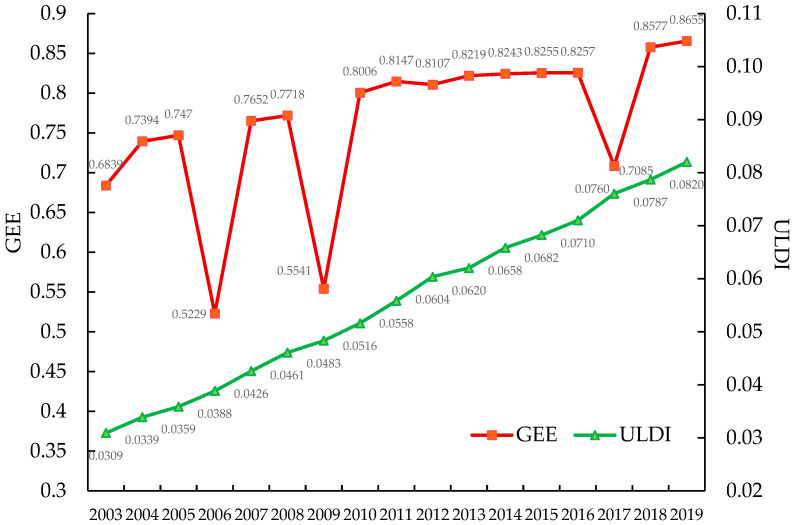
Calculation results and time-series trend characteristics of GEE and ULDI.

As can be seen from Figure 1, the ULDI increased year by year from 0.0309 in 2003 to 0.0820 in 2019. The measurement results show that during the study period, the overall ULDI in China showed a trend of gradual increase over time. The ULDI kept increasing with the rapid advancement of industrialization and urbanization. The GEE increased from 0.6839 in 2003 to 0.8655 in 2019. The measurement results show that during the study period, China’s GEE showed a trend of gradual improvement over time. However, the average green economic efficiency in each year has not reached the effective value. From the perspective of time series evolution, China’s GEE shows a staged characteristic of a steady rise in fluctuations. From 2003 to 2010, the change of green economic efficiency was dominated by “fluctuations, supplemented by rising” and green economic efficiency did not form a stable upward trend. From 2010 to 2016, GEE showed a steady upward trend. From 2016 to 2019, GEE showed an upward trend after a significant decline in 2017.

The evolutionary characteristics of ULDI and GEE show that both ULDI and GEE showed an upward trend from 2003 to 2019. It shows that the support efficiency of China’s urban land development to the improvement of green economic efficiency continues to increase, and urban land development tends to develop in a good operation and adaptation state. However, the changing trends of ULDI and GEE are different. During the sample period, the ULDI was in a relatively steady upward trend year by year, while the GEE showed a fluctuating upward trend of “decrease first and then increase”. When the GEE is on the rise, the increase in the ULDI is larger than that when the GEE is on the decline.

### 3.2. Analysis on the Interactive Response between ULDI and GEE

The PVAR is built in the following four steps [21,60]. First, we used the unit root to test the stationarity of the time-series data, and use the Granger method to test whether there is a causal relationship between GEE and ULDI. Second, we chose the suitable lag order and use the generalized moment method to determine the regression result among GEE and ULDI. The impulse response functions will be tested third.

#### 3.2.1. Stationarity and Causality Tests

Time-series variables need to be tested for their stationarity by unit root, and the PVAR model cannot predict the change law of nonstationary time-series data. The commonly used test methods of Levin-Lin-Chu (LLC), Im-Pesaran-Shin (IPS), and Fisher-type (ADF-Fisher, PP-Fisher) are comprehensively used. The results are shown in Table 3. As can be seen from Table 3, the *p*-values of the LLC, IPS, Fisher-ADF, and Fisher-PP test statistics of green economic efficiency and territorial space development intensity are all 0.0000, and the original data are all stationary data.

The Granger method is used to test the causality between ULDI and GEE. The results are shown in Table 4. As can be seen from Table 4, the *p*-values of the Granger causality test of ULDI for GEE and GEE for ULDI are both 0.000, indicating that the Granger null hypothesis is rejected. There is a two-way interactive Granger causality between ULDI and GEE.

#### 3.2.2. PVAR Model Regression Analysis

According to the constructed formula (9), we can use the Stata15.0 software to determine the regression result among GEE and ULDI. The implementing of the PVAR model first needs to determine the optimal lag order. Same as in the existing literature [21], we used Akaike’s information criterion (AIC), Baysian information criterion (BIC), and Hannan and Quinn information criterion (HQIC) to select the optimal lag order (Table 5). It can be found from Table 5 that when the lag order is 4, the statistics of AIC, BIC, and HQIC are the minimum. Therefore, 4 is the lag order selected to establish the PVAR (9) model.

We used the generalized method of moments (GMM) method to estimate the PVAR model [60]. The results are shown in Table 6, among which the ULDI equation under “Type” represents the effect of ULDI and GEE on ULDI, and the GEE equation under “Type” represents the effect of GEE and ULDI on GEE, and L1. represents the variable of the first-period lag, L2. represents the variable of the second-period lag, L3. represents the variable of the third-period lag, and L4. represents the variable of the fourth-period lag.

As shown in Table 6, in the ULDI equation, the L1., L2., L3., and L4. of both ULDI and GEE have positive influence coefficients on ULDI in the current period. However, the impact of the L1., L2., L3., and L4. of ULDI on the current is only significant in L1. ULDI generally has a large degree of path-dependent inertia in the subsequent development process, but because of the limitation of land resources, this path dependence gradually weakens over time. The positive effect of L1., L2., L3., and L4. of GEE on ULDI is consistently significant. The improvement of GEE is an important reason affecting ULDI and the impact of the improvement of GEE on ULDI has a positive cumulative effect on the time scale.

In the GEE equation shown in Table 6, the L1., L2., L3., and L4. of GEE have alternating positive and negative effects on GEE in the current period. GEE has a large degree of self-adjustment mechanism, and this self-adjustment is manifested as self-promotion. The L1., L2., L3., and L4. of ULDI also showed alternating positive and negative effects on GEE. The influence effect of ULDI on GEE has nonlinear characteristics, which is manifested as a divergent promoting effect on GEE in the early and middle stages of green economic development, and a convergent hindering effect in the short-term and late stages of green economic development. In the long run, the positive effect of ULDI on GEE is greater than the negative effect.

#### 3.2.3. Impulse Response Analysis

**Figure 2:**
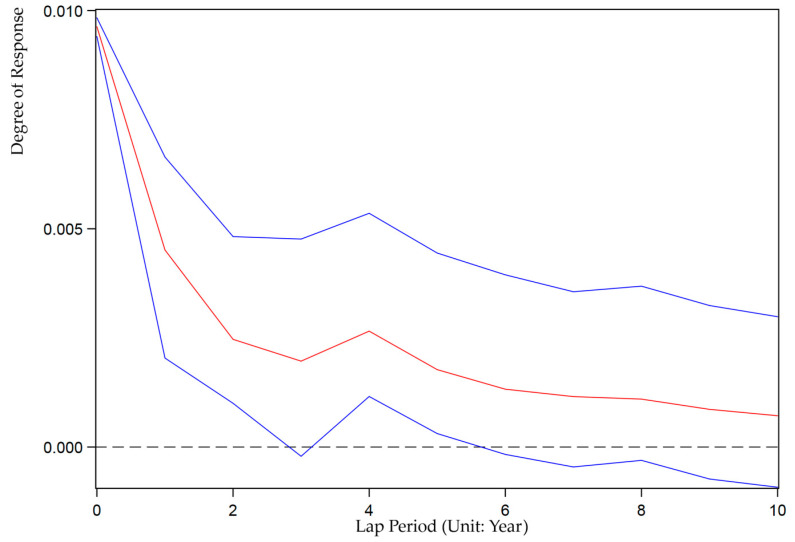
Impulse response of ULDI to ULDI.

**Figure 3:**
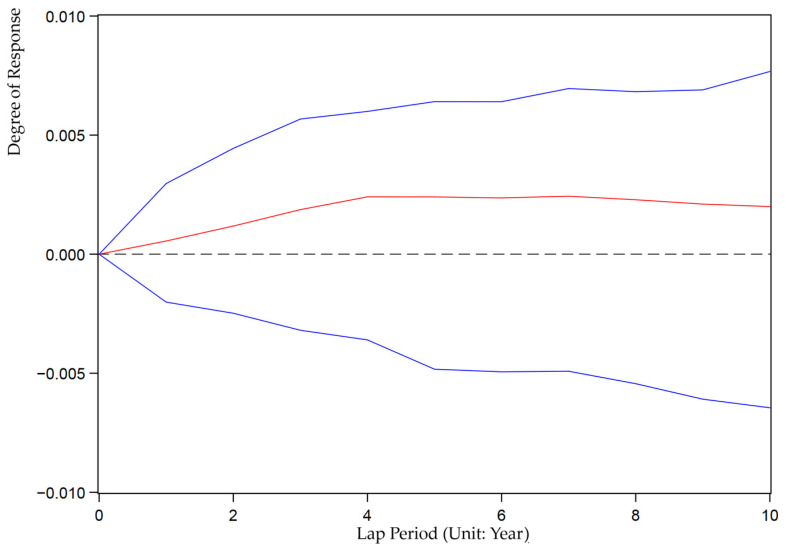
Impulse response of ULDI to GEE.

**Figure 4:**
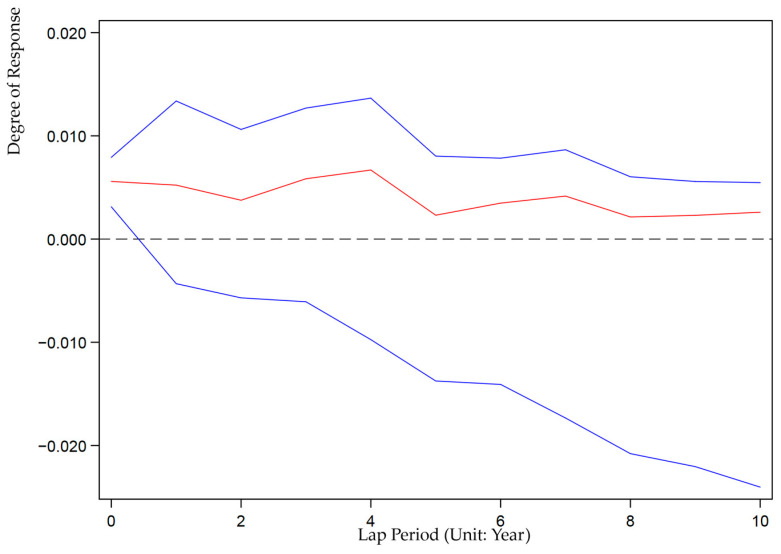
Impulse response of GEE to ULDI.

**Figure 5:**
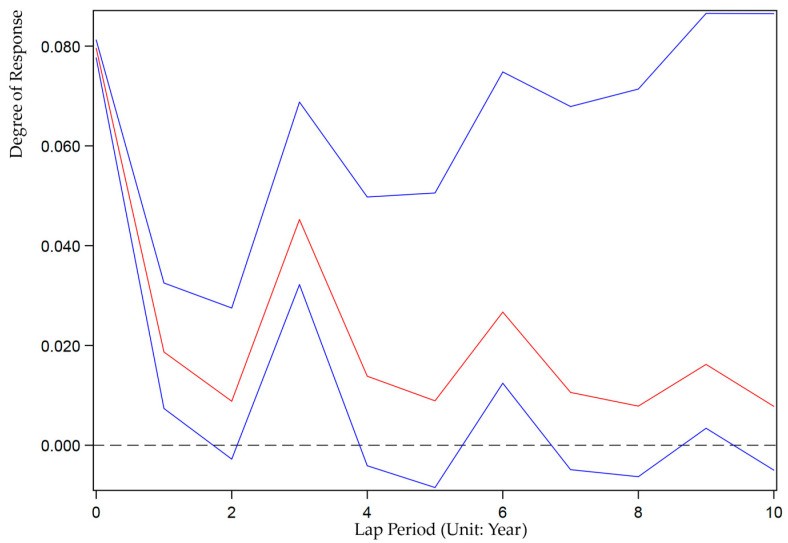
Impulse response of GEE to GEE.

We also use the Stata15.0 software based on Equation (9) to analyze the impulse response between ULDI and GEE. We set up 200 Monte Carlo simulations to investigate the impact of random disturbances under unit standard deviation on the dynamic evolution of current and future values of variables to reveal the interactive response mechanism of China’s ULDI and GEE in the next 10 years. Figure 2 and Figure 3 show the graph of the impulse response function of ULCI to itself and GEE. Figure 4 and Figure 5 show the graph of the impulse response function of GEE to itself and ULCI.

As shown in Figure 2, ULDI has a positive effect on the impulse response of its own unit, and the impact effect is severe. When it is impacted by its own unit standard deviation, ULDI quickly responds to the peak value in the current period, and then this positive response shows a fluctuating downward trend and converges to 0.

As shown in Figure 3, the impulse response of ULDI to GEE is positive. It shows that the current response is 0, and then the positive response speed is accelerated, reaching the highest value in the fourth period, and each period after the fourth period has a relatively stable positive response.

As shown in Figure 4, when subjected to one standard deviation shock of ULDI, GEE showed a positive response in the 10th period, but its impulse response showed periodic U-shaped fluctuations. It shows that ULDI has a volatile promoting effect on the improvement of GEE. ULDI has always been the driving force for the improvement of GEE, but in this process, it is necessary to continuously improve the efficiency of resource utilization and weaken the restrictive effect of urban land development on the improvement of GEE.

As shown in Figure 5, when subjected to its own unit standard deviation, GEE also showed a positive response. The specific performance is that it also responds quickly in the current period and reaches the peak value. After that, it showed the characteristics of reciprocating change, of which the positive effect decreases in the first period, tends to be weak in the second period, and increases in the third period. 

## 4. Discussion

This paper defines the ULDI including the urban construction land scale, the internal type of urban construction land, and the economic, social, ecological benefits of urban land development. It reflects the current important measures to realize the sustainable development of urban land development in China, that is, to control the disorderly expansion of the scale of construction land in breadth, to optimize the types of urban construction land through internal potential tapping to the limit, and to continuously improve the benefits of urban land development in depth. This provides a reference for other developing countries to pay attention to the ULDI in the process of development.

However, only the internal improvement of ULDI is effective in the short term, but unsustainable in the long run. The rapid growth of China’s economy depends on the direct pull of the wide supply of urban land. China is faced with the problem of insufficient capital in the early stage of economic development. China’s unique land system, with the compulsory low-cost land acquisition system and the government-monopolized state-owned land transfer system as the core arrangement, ensures a wide supply of land and makes land resource utilization a source of capital for economic development [61]. A large amount of low-cost supply of land has become an important tool for local governments to attract investment and obtain more investment in fixed assets, and promote the development of local industrialization [62,63]. Especially after the reform of the tax-sharing system, local governments have obtained a large amount of land transfer income and tax by increasing the supply of urban development land, bringing a large amount of land fiscal revenue [64]. Land fiscal revenue and land financing mortgage funds have become important sources of funds for local governments to realize infrastructure construction, and further attract the inflow of capital and talents [65]. Land has become a factor of production as important as technology, capital, and labor in the process of economic development, and urban land development as a tool has created a miracle of economic growth in China.

Many problems have begun to emerge from this traditional economic development model accumulated with the extension of the time domain and the frequency domain. First, the economic development model that relies too much on land resource utilization is unsustainable because of the real constraints of a scarcity of land resources. In addition, the development model of land capitalization in which local governments bundle land transfer fees and reserve land mortgage financing has accumulated a lot of financial risks [66]. These further inhibit economic growth and urbanization. Second, environmental problems such as carbon emissions and industrial pollutant emissions are significant. Local governments rely on low-cost land supply to attract a large number of low-end manufacturing industries with high energy consumption and high pollution, which has greatly promoted the process of industrialization. However, in the long run, enterprises with poor prospects and low production capacity squeeze land resources, and the “crowding out effect” of technology-intensive and capital-intensive high-value-added industries reduces industrial output value [67]. Third, bound by the law of diminishing marginal returns, the economic output that can be brought about by an increase in unit land investment is becoming more and more limited, and the engine function of land driving economic growth and regulating economic rhythm begins to decline [68]. Ultimately, China’s economy has to face the transformation of old and new economic growth drivers.

China chooses a green way of economic development. From 2003 to 2010, China transformed the traditional economic development model of high pollution and high energy consumption. During this period, China’s “Eleventh Five-Year Development” plan has placed emphasis and strategic arrangements on structural adjustment, energy conservation and emission reduction, and coordinated regional development. After comprehensively considering economic growth and resource environmental protection, the GEE has declined. From 2010 to 2016, with technological innovation, industrial restructuring, environmental regulation, and other measures, the GEE began to rise steadily. Since then, China’s green economy model has continued to make new achievements. Because there is a direct elastic mechanism of urban land development, unit land input will bring about an increase in output. Therefore, even if China’s economy completes the phased transformation of new and old kinetic energy, urban land development as a traditional economic growth kinetic energy still exists, and the scale of urban land development and its growth rate still needs to be maintained at a certain level. Moreover, the green economy development faces the dual goals of increasing total demand and improving efficiency [69]. The increase in aggregate demand for economic development will inevitably lead to and coerce an increase in aggregate supply, that is, an increase in total economic output will inevitably lead to an increase in the scale or marginal output of capital, labor, and land [70]. At the same time, urban land development faces the constraints of limited total land resources and conforms to the law of marginal diminishing returns to land. Only by ensuring the sustainability of ULDI can we achieve the level of total economic output and improve the GEE.

Therefore, our findings confirm that there is a mutual influence and mutual promotion between urban land development and green economic development. Land resources are an indispensable element of economic development. The driving effect of urban land development on economic development will not be significantly adjusted or changed. No matter what kind of economic development mode, the input of urban land resources is required. Under the green economic development model, the development of the green economy presents a strong self-adjustment mechanism, which can adjust itself according to the actual development situation to ensure the sustainable development of the green economy. In addition, through technological innovation, industrial structure transformation, and upgrading, etc. to improve the GEE, it can effectively promote the sustainability of urban land development and achieve a balance between urban land development and protection. To achieve sustainable development goals, whether it is for China or other developing countries, it is not sustainable to rely solely on the optimization within the urban land development system or within the economic development system, but to achieve a benign interaction and collaborative development between urban land development and green economy are promising.

## 5. Conclusions

This paper has explored the dynamic relationship between ULDI and GEE. It mainly draws the following two conclusions: (1) from 2003 to 2019, China’s ULDI and GEE showed a relatively obvious upward trend, and the increase in ULDI in the period of increasing GEE was larger than that in the period of declining GEE. The growth and evolution trend of ULDI and GEE has the characteristics of interaction and coordination. (2) There is a two-way interactive Granger causality between ULDI and GEE. The GMM model estimation results show that both ULDI and GEE have positive inertial growth and self-enhancement mechanisms. The interaction between GEE and ULDI has nonlinear characteristics, which is manifested as a positive cumulative effect on the time scale of the effect of GEE on ULDI, but the effect of ULDI on GEE only has a significant positive enhancement effect in the short term, but this contribution gradually weakened as the number of ULDI lag periods increased. From the results of impulse response analysis, ULDI has a positive response to GEE, which tends to be stable after reaching the highest value in the fourth period and has a significant positive enhancement effect in the long run. The results of impulse response analysis also showed that GEE also had a significant positive response to ULDI, and its impulse response showed a phased “U”-shaped fluctuation trajectory, and ULDI has a fluctuating promoting effect on GEE. 

When discussing sustainable development issues from the perspective of urban land use and economic development, it is different from looking at issues from one side. Our novel research perspective is to examine the bidirectional dynamic relationship between GEE and ULDI. The empirical test based on the interaction and response mechanism between GEE and ULDI provides a basis for realizing the path of sustainable development by realizing the urban land development system, the green economic development system, and promoting the good mutual feedback evolution between the two. It can be seen that the development of ULDI can play a positive role in improving GEE. With the expansion of construction land scale, the adjustment of land-use type structure and the improvement of urban land development functions, the green economy development can be continuously promoted. However, when the ULDI reaches a certain level, its influence on GEE will continue to weaken or even have a negative impact. However, at the same time, when GEE is improved through technological innovation, industrial structure transformation, and upgrading, it can continuously optimize the urban land development structure and improve the comprehensive benefits of urban land development, and reduce the dependence of economic development on urban land resources to a greater extent. Similarly, when GEE increases to a certain range, its impact on ULDI will continue to weaken and eventually stabilize. This means that under the support of a certain ULDI, GEE has been improved to a certain level, a new balance has been achieved between ULDI and GEE, and the whole society is in a state of a virtuous circle of sustainable development. However, simply relying on the internal optimization of the urban land development system to improve the ULDI or relying on the internal optimization of the green economic development system to improve GEE is not sustainable. Regional sustainable development plans and policies should be formulated from the perspective of the coordinated development of urban land development and green economy according to their own development conditions.

Admittedly, this study has several limitations. For example, first, there are other indicators and methods for measuring the status quo of urban land development and green economy development. Even the existing index system in this paper also needs to be further supplemented and improved according to the actual situation of each country or city. Second, this study only considers the interactive response between green economic efficiency and urban land development intensity. Studies have shown that resource endowment, population size, and structural characteristics, policies, and regulations have significant impacts on urban land development intensity and green economic efficiency. The current study does not incorporate these factors into the analytical framework. Third, there are significant regional heterogeneities in the resource endowment conditions and social and economic development levels of various countries, and there may be regional differences in the interactive response effect between green economic efficiency and the intensity of national land and space development. Future research will explore the construction of a more scientific index system and method for measuring urban land development intensity and green economic efficiency, and incorporate regional heterogeneity into the study of the relationship between the two. It is also a future research direction to refine the internal dimension of green economic efficiency improvement and the internal dimension of urban land development intensity to reveal the interactive response mechanism between green economic efficiency and urban land development intensity at a deeper level.

## Figures and Tables

**Table 1:** GEE evaluation index system.

Target	Index	Category	Indicator Explanation
GEE	input	Labor input	Number of employees (person)
		capital input	Fixed asset investment (RMB 10,000)
		Technology input	Number of green patents (pieces)
		Energy input	Electricity consumption of the whole society (100 million kWh)
	output	expected output	GDP (RMB 10,000)
		undesired output	Industrial wastewater discharge (tons)
			Industrial exhaust emissions (tons)
			Industrial solid waste discharge (tons)
			Carbon emissions (tons)

**Table 2:** ULDI evaluation index system.

Target	Category	Indicator Explanation
ULDI	The scale of urban land development	The ratio of construction land area to total land area in the region (%)
	Economic benefits of urban land development	Industrial non-agricultural rate (%)
		GDP output per land (10,000 RMB/square kilometer)
	Social benefits of urban land development	Per capita disposable income (yuan/person)
		Per capita residential land area (square meters/person)
		Per capita road area (square meters/person)
	Ecological benefit of urban land development	green space per capita (square meters/person)
	Urban land development structure	Information entropy of construction land structure

**Table 3:** Unit root test results.

Variables	LLC		IPS		Fisher-ADF		Fisher-PP	
	Statistics	*p*-Value	Statistics	*p*-Value	Statistics	*p*-Value	Statistics	*p*-Value
ULDI	−28.831	0.000	−10.505	0.000	6.524	0.000	24.153	0.000
GEE	−49.157	0.000	−34.443	0.000	47.999	0.000	75.619	0.000

**Table 4:** Granger causality test results.

	Null Hypothesis	Z-Bar Tilde	*p*-Value	Conclusion
ULDI	ULDI is not the Granger reason for GEE	27.690	0.000	Reject the null hypothesis
GEE	GEE is not the Granger reason for ULDI	15.706	0.003	Reject the null hypothesis

**Table 5:** Results of multi-criteria joint judgement.

Lag	AIC	BIC	HQIC
1	−2.317	−1.463	−2.015
2	−2.551	−1.641	−2.228
3	−2.811	−1.836	−2.464
4	−3.342	−2.291	−2.966
5	−3.308	−2.171	−2.900

**Table 6:** Estimation results of PVAR model based on GMM method.

Type	Variable	Coefficient	Variable	Coefficient
ULDI equation	L1.TSDI	0.504 (0.08) ***	L1.GEE	0.063 (0.02) ***
	L2.TSDI	0.049 (0.06)	L2.GEE	0.042 (0.02) **
	L3.TSDI	0.087 (0.06)	L3.GEE	0.070 (0.02) ***
	L4.TSDI	0.059 (0.05)	L4.GEE	0.032 (0.01) ***
GEE equation	L1.GEE	0.044 (0.01) ***	L1.TSDI	0.064 (0.03) **
	L2.GEE	−0.005 (0.01)	L2.TSDI	−0.008 (0.02)
	L3.GEE	0.470 (0.02) ***	L3.TSDI	0.016 (0.02)
	L4.GEE	−0.062 (0.01) ***	L4.TSDI	−0.006 (0.02)

Note: *** and ** show significance at the 1% level and 5% level, respectively. Std. Err. of estimated value is given in parentheses.

## Data Availability

Not applicable.
